# Bradyrhizobium cedriense sp. nov., a novel rhizobial species isolated from Acacia saligna nodules grown in polluted soils in Tunisia

**DOI:** 10.1099/ijsem.0.007127

**Published:** 2026-04-10

**Authors:** Nada Jihnaoui, Houda Zouagui, Jihed Hsouna, Takwa Gritli, Houda Ilahi, Fatma Souissi, Muhammad Sulman, Mustapha Missbah El Idrissi, Pierre Emmanuel Courty, Daniel Wipf, Hassen Gherbi, Walid Ellouze, Bacem Mnasri

**Affiliations:** 1 Laboratory of Legumes and Sustainable Agroecosystems, Centre of Biotechnology of Borj-Cédria, BP 901 Hammam-lif 2050, Tunisia; 2 Faculty of Sciences of Tunis, University Tunis El Manar, 2092 Tunis, Tunisia; 3 Microbiology and Molecular Biology Team, Center of Plant and Microbial Biotechnologies, Biodiversity and Environment, Faculty of Sciences, Mohammed V University in Rabat, Rabat, Morocco; 4 Agriculture and Agri-Food Canada, 4902 Victoria Avenue North, Vineland Station, Ontario L0R 2E0, Canada; 5 Faculty of Sciences, Centre de Biotechnologies Végétale et Microbienne, Biodiversité et Environnement, Mohammed V University in Rabat, Rabat, Morocco; 6 Agroécologie, Institut Agro Dijon, CNRS, Univ. Bourgogne, INRAE, Univ. Bourgogne Franche-Comté, F-21000 Dijon, France; 7 French National Research Institute for Sustainable Development (IRD), UMR PHIM (IRD/CIRAD/INRAe/Montpellier University/Institut Agro)- Campus International Baillarguet, TA A-120/J, 34398 Montpellier Cedex 5, France

**Keywords:** heavy metal tolerance, polyphasic taxonomy, phylogenomics, polluted soil, nitrogen fixation, rhizobia, symbiovar cyanophyllae

## Abstract

*Acacia saligna*, a leguminous plant known for its ability to thrive under challenging environmental conditions, plays an important role in bioremediation by enhancing soil fertility and facilitating pollutant degradation. In this study, three slow-growing rhizobial strains, designated 1.29L, 1.27L and 5.13L, were isolated from root nodules of *A. saligna* growing naturally in two contaminated sites in Tunisia. A polyphasic taxonomic approach was applied. Phylogenetic analysis based on the 16S rRNA gene placed the isolates within the genus *Bradyrhizobium*, clustering within the *Bradyrhizobium japonicum* superclade. Multilocus sequence analysis using concatenated housekeeping genes (*rec*A, *atp*D, *gln*II and *gyr*B: 1,734 bp) positioned the strains as a distinct lineage, with *Bradyrhizobium arachidis*, *Bradyrhizobium stylosanthis* and *Bradyrhizobium shewense* as the closest relatives, sharing 94.5–94.8% sequence identity. Comparative genomic analyses further supported their taxonomic distinctiveness. The proposed type strain, 1.29Lᵀ, exhibited average nucleotide identity (ANI) values of 90.6%, 90.1% and 89.1% with *Bradyrhizobium liaoningense*, *Bradyrhizobium diversitatis* and *Bradyrhizobium forestalis*, respectively, and digital DNA–DNA hybridization (dDDH) values ranging from 34.4 to 42.5%. In contrast, strains 1.29Lᵀ, 1.27L and 5.13L shared >99.9% ANI and 100% dDDH, confirming their conspecificity. Phylogenomic analyses conducted using the Type Strain Genome Server and GTDB-Tk pipelines further confirmed their placement within the *B. japonicum* supergroup while clearly separating them from all described species. Phenotypically, the isolates tolerated high concentrations of heavy metals and were capable of producing indole-3-acetic acid, solubilizing phosphate and producing siderophores. Based on the combined genomic, phylogenetic and phenotypic evidence, these strains represent a novel species, for which the name *Bradyrhizobium cedriense* sp. nov. is proposed, with strain 1.29Lᵀ (=LMG 33169ᵀ=DSM 116455ᵀ) designated as the type strain.

## Data Summary

The GenBank/EMBL/DDBJ accession for whole- genome sequences is JBTSWB000000000, JBTSWA000000000 and JBTSVZ000000000 with 16S rRNA (OR091471, PX614131, PX614132), atpD (PX517230, PX517231, PX517232), recA (PX517221, PX517222, PX517223), glnII (PX517224, PX517225, PX517226), gyrB (PX517227, PX517228, PX517229) and nodC (PX557011, PX557012, PX557013) for strains 1.29LT, 1.27L and 5.13L, respectively. Four supplementary tables and four supplementary figures have been deposited in the Figshare repository and are publicly available under DOI: 10.6084/m9.figshare.31511791 [1].

## Introduction

*Acacia saligna* (Labill.) H.L.Wendl. is a leguminous tree native to Australia that has been widely introduced into semiarid regions worldwide, including North Africa, where it has become invasive [2]. In Tunisia, *A. saligna* was introduced primarily for rangeland rehabilitation, erosion control and dune stabilization, and it is now well established across a range of edaphic and climatic conditions [3,7]. In addition, *A. saligna* has shown considerable potential for phytoremediation due to its adaptability to harsh environments, including drought-prone, saline and alkaline soils [8,9]. The ecological success of *A. saligna* in these environments has been attributed, in part, to its capacity to establish effective nitrogen-fixing symbioses with diverse rhizobial taxa.

Previous studies have demonstrated that *A. saligna* is nodulated by a wide range of both fast- and slow-growing rhizobia [10]. In Tunisia, several rhizobial lineages associated with *A. saligna* have been identified, including strains affiliated with the *Rhizobium leguminosarum* complex, *Rhizobium acaciae*, *Rhizobium aouanii* [10,12] and *Bradyrhizobium tunisiense* [13]. In addition, two symbiovars nodulating *A. saligna* have been described: sv. cyanophyllae, affiliated with the genus *Bradyrhizobium*, and sv. *salignae*, affiliated with the *R. leguminosarum* complex [10]. These findings indicate a high degree of symbiotic diversity and suggest that *A. saligna* may act as a host for distinct and previously uncharacterized rhizobial taxa.

In the present study, three rhizobial strains, designated 1.29L, 1.27L and 5.13L, were isolated from root nodules of *A. saligna* plants collected in Tunisia. These strains were selected for detailed taxonomic analysis based on their symbiotic performance and high tolerance to heavy metals, traits that are relevant to plant establishment and phytoremediation potential. A comprehensive polyphasic taxonomic approach was employed, integrating whole-genome sequencing, phylogenomic analyses based on conserved core genes, comparative genomic metrics and detailed phenotypic and physiological characterization.

Phylogenomic analyses placed strains 1.29L, 1.27L and 5.13L within the genus *Bradyrhizobium*, forming a distinct and well-supported clade clearly separated from all currently described *Bradyrhizobium* species. Genomic relatedness indices, together with phenotypic differentiation from their closest phylogenetic relatives, supported their assignment to a novel species. Based on this collective polyphasic evidence, strains 1.29L, 1.27L and 5.13L are proposed to represent a novel species, for which the name *Bradyrhizobium cedriense* sp. nov. is proposed.

## Methods

### Isolation and ecology

The novel *Bradyrhizobium* strains 1.27L and 1.29L were isolated from surface-sterilized root nodules of *A. saligna* collected along the banks of Oued Soltane stream in northern Tunisia (36° 42′ 40.76″ N 10° 25′ 46.47″ E), a site impacted by industrial wastewater contamination with heavy metals. Strain 5.13L was isolated from root nodules of *A. saligna* collected along the contaminated stream Oued Hamdoun in central Tunisia (35° 45′ 36.18″ N 10° 38′ 35.08″ E). Isolation was performed following the protocol described by Vincent [14], with subsequent purification achieved by repeated streaking on yeast mannitol agar (YMA) as described by Hsouna *et al*. [10]. Cultures were incubated at 28 °C for 6 days, after which single colonies were selected and sub-cultured to ensure purity.

The type strain, 1.29L, has been deposited in two international culture collections: the BCCM/LMG Bacteria Collection (Ghent University, Belgium, accession number LMG 33169) and the DSMZ-German Collection of Microorganisms and Cell Cultures (Leibniz Institute, Germany, accession number DSM 116455).

### Phylogenetic analyses

The 16S rRNA gene, concatenated housekeeping genes (*atp*D, *rec*A, *gln*II and *gyr*B) and the symbiotic gene *nod*C were used for phylogenetic analyses. Sequence similarities were assessed using the blast program (https://blast.ncbi.nlm.nih.gov/Blast.cgi), and reference sequences were retrieved from GenBank National Center for Biotechnology Information (NCBI). Multiple sequence alignments were performed using clustalw2 (https://www.ebi.ac.uk/Tools/clustalw2/). Phylogenetic trees based on the 16S rRNA gene, the *nod*C gene and the concatenated *atp*D*-gln*II*-rec*A*-gyr*B gene sequences were reconstructed using the maximum-likelihood (ML) method implemented in mega version 12 [15]. Evolutionary distances were calculated using the Kimura two-parameter model [16], and branch support was evaluated by bootstrap analysis with 1,000 replicates.

### Genomic relatedness and phylogenomics

Whole-genome sequencing of the three novel strains, 1.27L, 1.29L and 5.13L, was performed using Illumina NovaSeq 6000 technology at Génome Québec (Montreal, Canada). Raw paired-end reads (2×150 bp) were assessed for quality using FastQC v0.11.9 [17]. Adapter and quality trimming were carried out using BBDuk from the BBTools package v39.01 [18]. Adapter sequences were removed by k-mer matching against a custom Illumina NovaSeq adapter reference, with right-end trimming enabled. Low-quality bases were trimmed from both 5′ and 3′ ends at a Phred score threshold of 20, and reads shorter than 50 bp were discarded. Sequence coverage was normalized to a maximum depth of 150× using BBNorm from the BBTools package v39.01 [18]. *De novo* genome assemblies were generated using SPAdes v3.15.5 [19] in careful mode. Assembly quality was evaluated with QUAST [20], as implemented in PATRIC v3.6.12 and the Bacterial and Viral Bioinformatics Resource Center (v3.42.3) [21,23]. Contigs shorter than 300 bp were excluded from subsequent analyses. Genome annotation was performed using GeneMarkS-2+ [24] within the NCBI Prokaryotic Genome Annotation Pipeline [25]. Summary statistics for the genome assemblies are provided in Table 1.

**Table 1.** Genome assembly and annotation statistics of the three strains of *B. cedriense* sp. nov. obtained using Illumina NovaSeq 6000 sequencing

Description	1.29L^T^	1.27**L**	5.13**L**
GenBank accession number	JBTSWB000000000	JBTSWA000000000	JBTSVZ000000000
Number of short paired-end reads (150 bp)	26.087	26.362	23.533
Average coverage depth	894	904	817
Contig count	49	26	60
Largest contig (bp)	1.315.855	1.599.741	1.442.886
Total length (bp)	7.878.461	7.878.339	7.796.323
Contigs N_50_ (bp)	977.975	1.147.892	669.946
Contigs L_50_	4	3	4
Total number of genes	7,345	7,328	7,307
Total coding sequences	7,289	7,272	7,252
Non-coding RNAs	3	3	3
rRNA: 5S, 16S, 23S	1, 1, 1	1, 1, 1	1, 1, 1
Number of tRNA	52	52	51
G+C content (mol%)	63.8	63.8	63.9

To determine the taxonomic position of strains 1.27L, 1.29L and 5.13L among described *Bradyrhizobium* species, average nucleotide identity (ANI) values were calculated against 80 *Bradyrhizobium* type strains using OrthoANIu v1.2 [26]. Digital DNA–DNA hybridization (dDDH) values were estimated using the Genome-to-Genome Distance Calculator v3.0 [27,28]. Phylogenomic relationships with closely related taxa were further inferred using the Type Strain Genome Server (TYGS). In addition, ANI comparisons were conducted between strain 1.29Lᵀ and a total of 1,412 publicly available *Bradyrhizobium* genome assemblies, followed by 329 metagenome-assembled genomes (MAGs) retrieved from GenBank as of 22 November 2025. These analyses were performed using FastANI v1.34 [29].

A complementary genome-based taxonomic classification of strains 1.27L, 1.29L and 5.13L was performed using the Genome Taxonomy Database (GTDB) release RS220 [30] and the GTDB-Tk v2.4.0 toolkit [31]. ANI values within the GTDB framework were estimated using skani v0.2.2 [32]. Gene prediction was carried out with Prodigal v2.6.3 [33], and homologues of 120 conserved bacterial marker genes were identified using HMMER v3.4 [34]. ML placement of the isolates within the GTDB reference tree was performed using pplacer v1.1 [35]. Finally, a *de novo* approximately ML phylogenomic tree of the genus *Bradyrhizobium* was reconstructed using FastTree v2.1.11 [36], based on a concatenated alignment of 116 conserved core bacterial markers (Table S1, available in the online Supplementary Material). The LG substitution model [37] was applied, and members of the genus *Nitrobacter* were used as the outgroup.

### Physiology, chemotaxonomy, heavy metal resistance and plant growth-promoting activities

Phenotypic and biochemical characteristics of the three novel strains were compared with those of closely related *Bradyrhizobium* species. Colony morphology was examined on YMA. Growth characteristics were evaluated in yeast extract mannitol (YEM) broth under different temperatures, NaCl concentrations [0.2%, 0.4%, 0.6%, 0.8% and 1.0% (w/v)] and pH levels ranging from 4.0 to 10.0 at 1.0-unit intervals. Antibiotic resistance was assessed on YEM agar plates supplemented with kanamycin, neomycin, ampicillin, erythromycin or penicillin at final concentrations of 5, 10, 20, 50 and 80 µg ml⁻¹. All assays were performed in triplicate. Carbon-source utilization and chemical sensitivity profiles were determined using Biolog GEN III MicroPlates according to the manufacturer’s instructions. Cellular fatty acid composition was analysed using the MIDI Sherlock Microbial Identification System (version 6.1) with the TSBA6 database. Resistance to heavy metals was evaluated by culturing the strains on tryptone–yeast extract (TY) agar plates supplemented with increasing concentrations (µg ml⁻¹) of ZnSO₄ (50, 100, 400, 600, 800 and 1,000), PbCl₂ (100, 300, 500, 800 and 1,000), CdCl₂ (10, 20 and 30) and CuSO₄ (50, 100, 200 and 300), as described by Lamrabet *et al*. [38]. Growth on TY agar in the presence of each metal was considered indicative of resistance.

Plant growth-promoting traits were evaluated following the methods described by Ilahi *et al*. [39]. Phosphate solubilization was assessed by inoculating the strains onto Pikovskaya agar supplemented with tricalcium phosphate and examining the formation of clear haloes around colonies after 6 days of incubation at 28 °C. Halo diameters were measured using Fiji software. Indole-3-acetic acid (IAA) production was quantified after growth in DF minimal medium supplemented with l-tryptophan, using a colourimetric assay with Salkowski’s reagent and comparison to a standard curve. Utilization of 1-aminocyclopropane-1-carboxylate (ACC) was assessed by incubating the strains in DF medium containing ACC and quantifying residual ACC using the ninhydrin reagent. ACC utilization was determined by comparison with a standard curve, and isolates were classified according to their ability to degrade ACC.

### Genome mining of the type strain 1.29L^T^ of *B. cedriense* sp. nov.

The genome of strain 1.29ᵀ was annotated with the Rapid Annotation using Subsystem Technology server [40], and biosynthetic gene clusters were identified with the Antibiotics and Secondary Metabolite Analysis Shell (antiSMASH) v6.0 [41]. The annotated genome was subsequently screened for genes putatively involved in plant growth-promoting functions and heavy metal resistance.

## Results and discussion

### Phylogenetic analyses

ML phylogenetic analysis based on partial 16S rRNA gene sequences (1,230 bp) confirmed that strains 1.29Lᵀ, 1.27L and 5.13L belong to the genus *Bradyrhizobium*, clustering within the *Bradyrhizobium japonicum* superclade (Fig. S1). Strain 1.29Lᵀ exhibited very high sequence similarity (99.93%) to *B. japonicum* USDA6ᵀ, originally isolated from soybean nodules in Japan [42]. The three strains also shared 99.85% 16S rRNA gene sequence similarity with *Bradyrhizobium liaoningense* LMG 18230ᵀ isolated from soybean nodules in Liaoning, China [43], as well as with *Bradyrhizobium diversitatis* CNPSo 4019ᵀ isolated from soybean nodules in Australia [44] and *Bradyrhizobium symbiodeficiens* 85S1MBᵀ, a non-symbiotic species associated with legumes in Canada [45].

Although the 16S rRNA gene remains a cornerstone of bacterial systematics, its high level of conservation within the genus *Bradyrhizobium* limits its resolving power at the species level [10,13]. As a result, species identification based solely on 16S rRNA gene analysis is often inadequate in the genus *Bradyrhizobium* [46,47]. To overcome this limitation, multilocus sequence analysis (MLSA) of housekeeping genes has been widely adopted, providing improved resolution and more reliable species delineation within *Bradyrhizobium* [10,48].

ML phylogenetic analysis based on concatenated housekeeping gene sequences (*rec*A, *gln*II, *atp*D and *gyr*B) clearly separated strains 1.29Lᵀ, 1.27L and 5.13L from all currently described *Bradyrhizobium* type strains (Fig. S2). The three strains displayed 100% sequence identity to one another and formed a well-supported monophyletic clade (bootstrap value=100%). Pairwise sequence similarity analysis showed that strain 1.29Lᵀ shared its highest MLSA similarity with *Bradyrhizobium arachidis* (94.76%), followed by *Bradyrhizobium stylosanthis* (94.70%) and *Bradyrhizobium shewense* (94.52%). These species were originally isolated from root nodules of *Arachis hypogaea*, *Stylosanthes* spp. (a tropical perennial forage legume) and *Erythrina brucei*, respectively [49,51]. Collectively, these results indicate that strain 1.29Lᵀ and its conspecific strains belong to the *B. japonicum* superclade but represent a clearly distinct phylogenetic lineage consistent with species-level differentiation.

### Genomic relatedness and phylogenomics

Draft genome sequencing of strains 1.29Lᵀ, 1.27L and 5.13L was performed using the Illumina NovaSeq platform, generating more than 30 million raw paired-end reads per strain. After quality filtering and normalization, the datasets used for assembly yielded mean genome coverage depths of 894×, 904× and 817× for strains 1.29Lᵀ, 1.27L and 5.13L, respectively. The assemblies were highly contiguous, with N50 values of 977,975 bp for strain 1.29Lᵀ, 1,147,892 bp for strain 1.27L and 669,946 bp for strain 5.13L, and corresponding L50 values of 4, 3 and 4. The total lengths of the draft genomes were 7,878,461 bp (1.29Lᵀ), 7,878,339 bp (1.27L) and 7,796,323 bp (5.13L), with G+C contents ranging from 63.8 to 63.9% (Table 1).

Genome annotation identified 7,345 genes in strain 1.29Lᵀ, 7328 in strain 1.27L and 7307 in strain 5.13L, including 7,289, 7,272 and 7,307 protein-coding sequences, respectively. Each genome encoded three rRNA operons (5S, 16S and 23S rRNAs). Fifty-two tRNA genes were detected in strains 1.29Lᵀ and 1.27L, while 51 tRNAs were identified in strain 5.13L. In addition, three non-coding RNAs were present in each genome (Table 1).

Genome-based taxonomic comparisons using ANI and dDDH were conducted against 80 validly published *Bradyrhizobium* type strains (Table 2). Strain 1.29Lᵀ exhibited ANI values ranging from 99.53 to 99.99% and dDDH values from 99.6 to 100% with strains 1.27L and 5.13L, clearly exceeding the recommended species delineation thresholds of 95–96% ANI and 70% dDDH. These results demonstrate that strains 1.29Lᵀ, 1.27L and 5.13L belong to the same genomic species. In contrast, all recognized *Bradyrhizobium* species showed ANI and dDDH values below the accepted species boundaries when compared with strain 1.29Lᵀ. OrthoANIu values ranged from 76.8 to 90.6%, while *in silico* dDDH estimates varied between 21.3 and 42.5% (Table 2), based on comparisons with reference *Bradyrhizobium* genomes available in GenBank. Among the closest relatives, *B. liaoningense* NBRC 100396ᵀ exhibited the highest relatedness, with an ANI value of 90.6% and a dDDH estimate of 42.5%. *B. diversitatis* CNPSo 4019ᵀ and *Bradyrhizobium forestalis* INPA54Bᵀ followed, showing ANI values of 90.1% and 89.1% and dDDH values of 41.1% and 38.6%, respectively (Table 2). These values are well below the species-level thresholds and are consistent with the distinct clustering observed in the *rec*A-*atp*D-*gyr*B-*gln*II MLSA phylogeny.

**Table 2.** ANI and dDDH values between *B. cedriense* sp. nov. strain 1.29Lᵀ (type strain) and related *Bradyrhizobium* species

*Bradyrhizobium* species	ANI (%)			dDDH (%)		
	1.27L	1.29L^T^	5.13L	1.27L	1.29L^T^	5.13L
***B. cedriense* sp. nov. 1.27L**	100	99.9	99.5	100.	100	96.6
***B. cedriense* sp. nov. 1.29L^T^**	99.9	100	99.5	100	100	96.6
***B. cedriense* sp. nov. 5.13L**	99.5	99.5	100	96.6	96.6	100
*B. liaoningense* NBRC100396^T^ (GCA_030160735.1)	90.6	90.6	90.6	42.5	42.5	42.5
*B. diversitatis* CNPSo4019^T^ (GCA_016031635.1)	90.1	90.1	90.3	41.1	41.1	41.4
‘*B. forestalis*’ INPA54B^T^ (GCA_002795245.1)	89	89.1	89.1	38.6	38.6	38.4
*B. vignae* LMG28791^T^ (GCA_004114425.1)	88.4	88.4	88.5	37.1	37	37
*B. glycinis* CNPSo4016^T^ (GCA_016031655.1)	88.3	88.3	88.2	36.4	36.4	36.3
*B. agreste* CNPSo4010^T^ (GCA_016031625.1)	88.1	88.1	88.1	36.2	36.2	36.2
*B. tunisiense* 1AS2L^T^ (GCA_042263485.1)	88.1	88.1	87.9	35.4	35.4	35.1
*B. yuanmingense* CCBAU10071^T^ (GCA_900094575.1)	87.9	88	88.1	36	36	36.2
‘*B. zhanjiangense*’ CCBAU51778^T^ (GCA_004114935.1)	87.8	87.7	87.9	35.9	35.8	35.7
*B. shewense* ERR11^T^ (GCA_900094605.1)	87.7	87.6	87.5	35	35	34.9
*B. xenonodulans* 14AB^T^ (GCA_027594865.1)	87.7	87.7	87.6	34.8	34.8	34.4
*B. cajani* 1010^T^ (GCA_009759665.1)	87.7	87.7	87.6	35.3	35.3	35.3
*B. ottawaense* OO99GCA^T^ (002278135.3)	87.7	87.7	87.8	35.2	35.2	35.2
*B. frederickii* CNPSo3426^T^ (GCA_004570865.1)	87.7	87.7	87.8	35.4	35.4	35.3
*B. nitroreducens* TSA1^T^ (GCA_002776695.1)	87.7	87.6	87.7	34.4	34.4	34.5
*B. symbiodeficiens* 85S1MB^T^ (GCA_002266465.3)	87.6	87.6	87.6	34.3	34.3	34.3

**Fig. 1.**
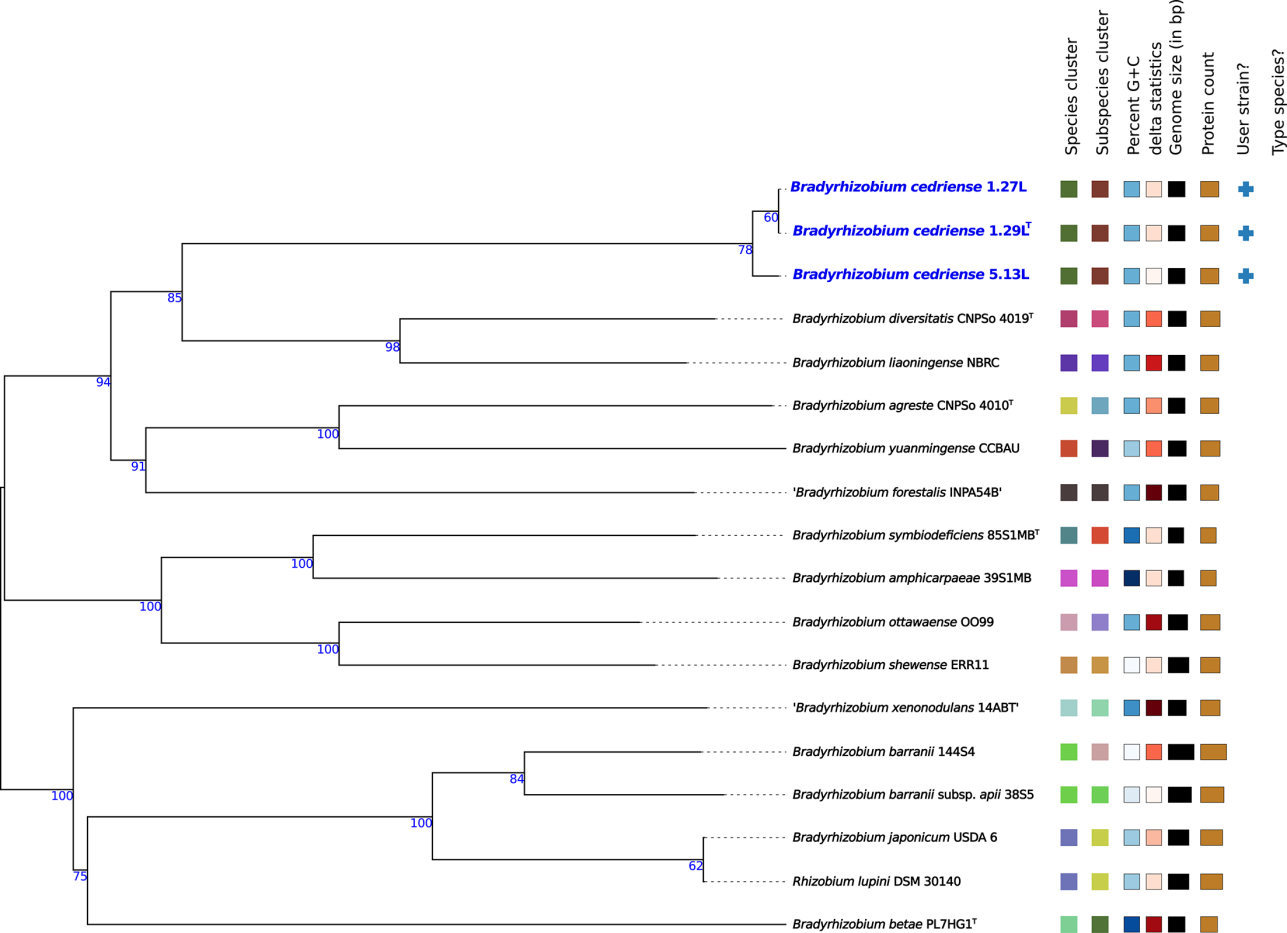
Balanced minimum-evolution tree based on intergenomic distances among strains 1.27L, 1.29L, 5.13L and the closest type strains of the genus *Bradyrhizobium*. Branch support was evaluated using 100 pseudo-bootstrap replicates. The tree was rooted at the midpoint.

The phylogenomic tree inferred using the TYGS further supported the genomic distinctiveness of strains 1.29Lᵀ, 1.27L and 5.13L, which formed a well-supported and clearly separated lineage within the genus *Bradyrhizobium* (Fig. 1). To evaluate the presence of closely related uncultured or unclassified genomes, FastANI comparisons were performed between strain 1.29Lᵀ and 1,741 publicly available genome sequences, including 1,412 isolate genomes and 329 MAGs affiliated with *Bradyrhizobium*. All ANI values obtained were below the accepted species threshold (Table S2), indicating that none of the available genomes correspond to the proposed novel species.

**Fig. 2.**
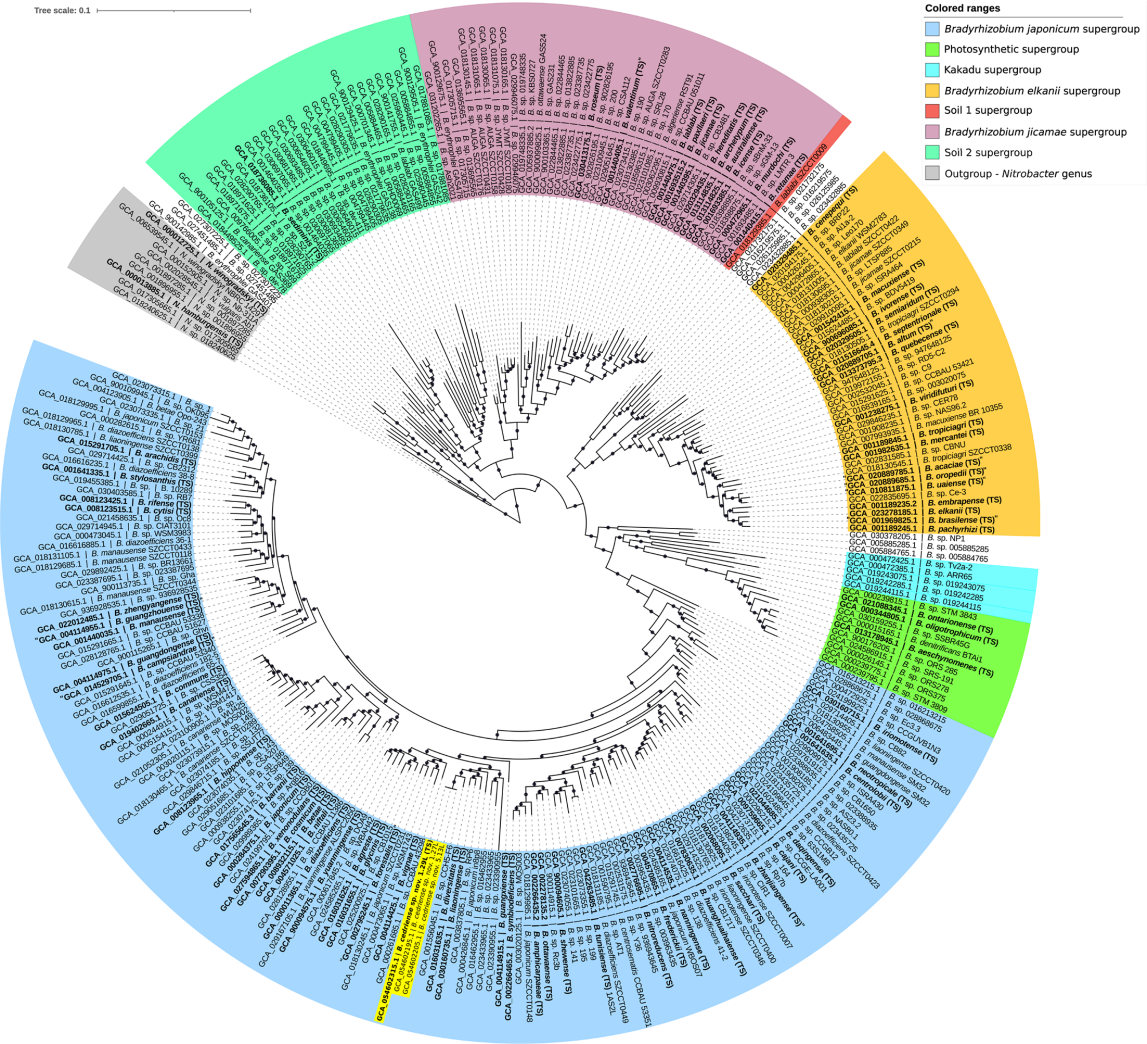
*De novo* approximately ML phylogenomic tree of the genus *Bradyrhizobium* inferred using GTDB reference genomes, with the novel strains highlighted in yellow. The tree was reconstructed using 116 conserved bacterial marker genes and rooted with members of the genus *Nitrobacter*. Local support values ≥70% are indicated by circles on the corresponding branches (based on 1,000 replicates). Type strains are shown in bold. An ANI threshold of 96% was applied for strain delineation. The scale bar represents the number of substitutions per site.

**Fig. 3.**
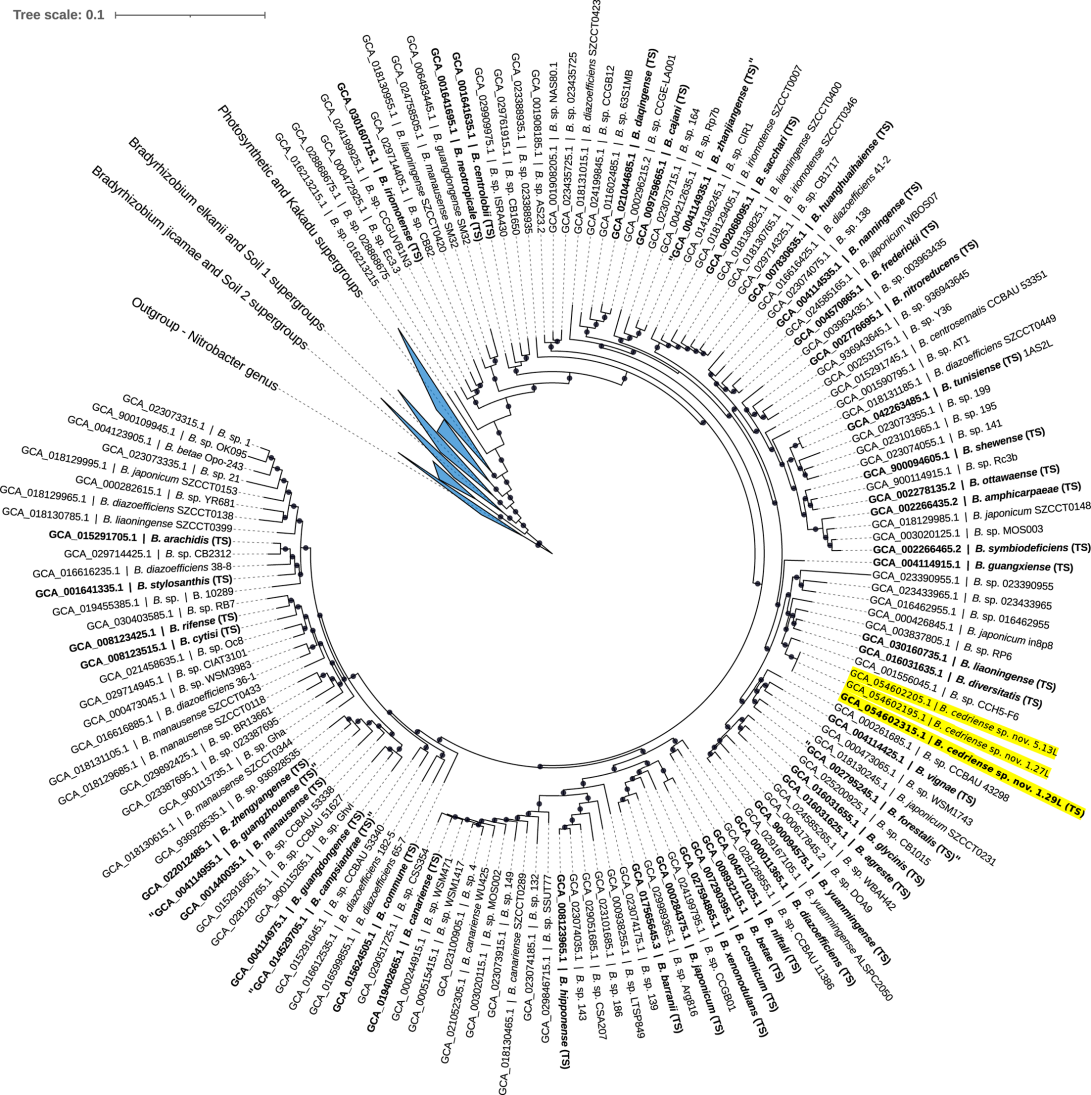
*De novo* approximately ML phylogenomic tree of the genus *Bradyrhizobium* focusing on the *B. japonicum* supergroup, inferred using GTDB reference genomes. The novel strains are highlighted in yellow. The tree was reconstructed using 116 conserved bacterial marker genes and rooted with members of the genus *Nitrobacter*. Local support values ≥70% are indicated by circles on the corresponding branches (based on 1,000 replicates). Type strains are shown in bold. Peripheral supergroups were collapsed to improve the visualization of the studied cluster. The scale bar represents the number of substitutions per site.

Consistent results were obtained using the GTDB framework. Based on ANI estimates and ML placement, strains 1.29Lᵀ, 1.27L and 5.13L were assigned to the genus *Bradyrhizobium* but could not be affiliated with any validly published species. The *de novo* phylogenomic reconstruction revealed that most reference genomes clustered into seven previously defined supergroups within the genus [52]. The three strains were positioned within the *B. japonicum* supergroup (Fig. 2). For clarity, peripheral branches of the tree were collapsed. Strains 1.29Lᵀ, 1.27L and 5.13L formed a highly supported monophyletic clade, with *B. liaoningense* and *B. diversitatis* forming a closely related sister clade (Fig. 3). Collectively, these genome-based analyses conclusively support the proposal that strains 1.29Lᵀ, 1.27L and 5.13L represent a novel species within the genus *Bradyrhizobium*.

### Symbiotic analyses

**Fig. 4.**
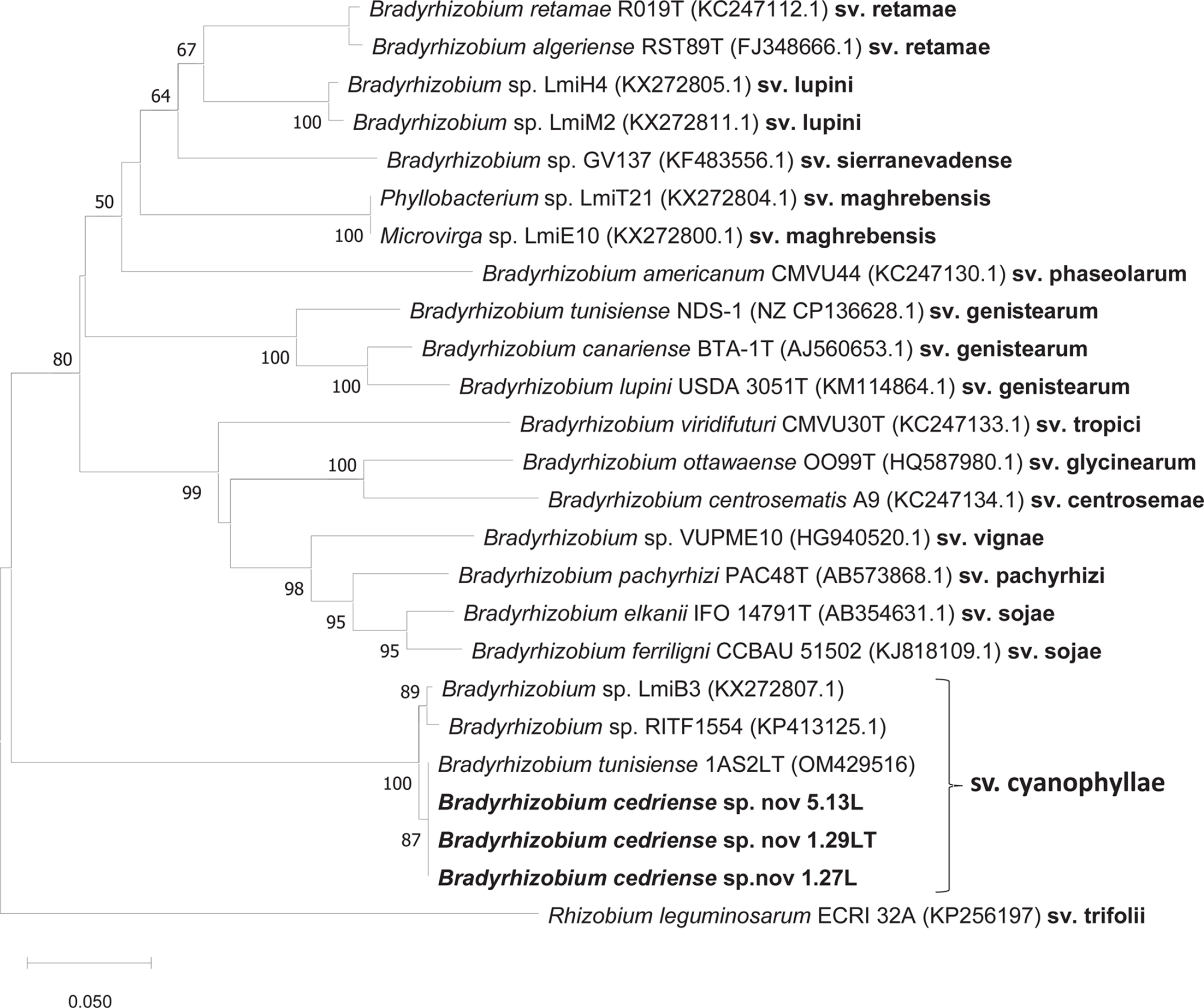
ML phylogenetic tree based on *nodC* gene sequences (377 nt) illustrating symbiovar relationships within the genus *Bradyrhizobium*. Isolates of *B. cedriense* sp. nov. are highlighted in bold. Bootstrap values ≥50% are shown at the corresponding nodes (based on 1,000 replicates). The scale bar represents the number of substitutions per site.

The *nod*C gene is widely used for symbiovar assignment in rhizobia. In the present study, phylogenetic analysis of *nodC* sequences revealed that the strains of *B. cedriense* sp. nov. form a distinct symbiotic group (Fig. 4). This group comprises the *B. cedriense* strains together with the reference strain *B. tunisiense* 1AS2Lᵀ, all of which were isolated from root nodules of *A. saligna* [13]. Based on *nodC* phylogeny, these strains were assigned to the symbiovar *cyanophyllae*.

Nodulation tests were performed using *A. saligna* as the host plant. The three strains, including the proposed type strain 1.29Lᵀ, were individually inoculated onto sterile *A. saligna* seedlings under controlled conditions. All three strains successfully induced nodules, and effective nodulation was confirmed by the presence of well-developed nodules with characteristic pink internal coloration, indicating active nitrogen fixation.

Previous studies have shown that members of this symbiovar are highly effective in nodule formation and nitrogen fixation with several host plants, including *A. saligna*, *Acacia salicina*, *Leucaena leucocephala* and *Acacia tortilis* [10]. However, nitrogen-fixing efficiency varies among host species. For example, although these strains are able to nodulate *Phaseolus vulgaris*, they do not fix nitrogen in this host. In addition, they fail to nodulate *Glycine max* (soybean) and *Retama raetam*.

### Physiology and chemotaxonomy

Phenotypic and chemotaxonomic characteristics of strains 1.29Lᵀ, 1.27L and 5.13L were examined in comparison with the type strains *B. liaoningense* LMG 18230ᵀ, *B. diversitatis* CNPSo 4019ᵀ, *Bradyrhizobium glycinis* CNPSo 4016ᵀ and *B. tunisiense* 1AS2Lᵀ. These taxa were selected based on their relatively high ANI and dDDH values with strain 1.29Lᵀ, although all values remained below the accepted species delineation thresholds (Table 3).

**Table 3.** Phenotypic characteristics determined using Biolog GEN III MicroPlates for strains *B. cedriense* sp. nov. 1.29Lᵀ, 5.13L and 1.27L, (1) *B. diversitatis* CNPo4019ᵀ, (2) *B. liaoningense* LMG 18230ᵀ, (3) *B. glycinis* CNPS 04016ᵀ and (4) *B. tunisiense* 1AS2Lᵀ

Biolog substrate	1.29L^T^	5.13**L**	1.27**L**	1	2	3	4
d-Maltose	−	−	−	w	−	w	−
d-Trehalose	−	−	−	w	w	w	−
d-Cellobiose	−	−	−	w	−	w	−
Gentiobiose	−	−	−	w	−	w	−
Sucrose	−	−	−	w	−	w	−
d-Turanose	−	−	−	w	−	+	−
d-Raffinose	−	−	−	w	−	w	−
*α*-d-Lactose	−	−	−	w	−	w	−
d-Melibiose	−	−	−	w	−	w	−
d-Salicin	−	−	−	w	−	w	−
d-Fructose	+	+	+	−	−	w	−
d-Galactose	+	+	+	−	w	w	−
d-Fucose	+	+	+	w	+	+	+
l-Rhamnose	−	−	−	w	w	w	−
d-Sorbitol	+	+	+	w	−	w	−
d-Mannitol	+	+	+	w	−	w	−
d-Arabitol	−	−	−	w	w	w	
Myo-inositol	−	−	−	−	−	w	−
Glycerol	+	+	+	w	w	w	+
d-Glucose-6-PO4	−	−	−	w	w	w	+
d-Fructose-6-PO4	−	−	−	−	−	+	+
**Chemical sensitivity**							
pH 10	−	−	−				
pH 5	+	+	+	+	+	+	
NaCl 0.8	+	+	+				−
NaCl 1%	−	−	−	−	−	−	
**Tolerance to antibiotics**							
Ampicillin (80 µg ml^−1^)	+	+	+	−	−	−	−
Neomycin (20 µg ml^−1^)	−	−	−	+	−	w	+
Streptomycin (50 µg ml^−1^)	+	+	+	−	−	−	+
Erythromycin (50 µg ml^−1^)	+	+	+	+	+	−	−

Results are recorded as follows: +, positive; −, negative; w, weakly positive.

Cells of strains 1.29Lᵀ, 1.27L and 5.13L were Gram-negative, aerobic, non-spore-forming rods. Colonies on YEM agar were circular, convex, smooth and pink after 6 days of incubation at 28 °C. Scanning electron microscopy revealed rod-shaped cells ~1.8 µm in length and 0.7 µm in width (Fig. S3). The strains were salt-sensitive and did not grow in the presence of NaCl concentrations exceeding 1% (w/v). Growth occurred at pH values between 6.0 and 9.0 and at temperatures ranging from 18 to 37 °C, with optimal growth observed at 28 °C and pH 7.0, consistent with reported physiological traits of members of the genus *Bradyrhizobium* [13,53].

Carbon-source utilization patterns were identical among the three strains of *B. cedriense* sp. nov. and allowed their differentiation from at least one of the closest related type strains. The strains assimilated d-fructose, d-galactose, d-fucose, d-sorbitol, d-mannitol and glycerol (Table 3). In contrast, none of the three strains of *B. cedriense* sp. nov. utilized d-maltose, d-trehalose, d-cellobiose, gentiobiose, sucrose, d-turanose, d-raffinose, d-lactose, d-melibiose, d-salicin, l-rhamnose, d-arabitol, myo-inositol, d-fructose-6-phosphate or d-glucose-6-phosphate.

The type strain 1.29Lᵀ was resistant to ampicillin (80 µg ml⁻¹), streptomycin (50 µg ml⁻¹) and erythromycin (50 µg ml⁻¹), but sensitive to neomycin. Cellular fatty acid analysis revealed that the predominant fatty acid was summed feature 8 (C_18 : 1_ ω7c) (53.0%), followed by C_18 : 2_ ω6 (17.3%) and C_16 : 0_ (15.9%), a profile consistent with other members of the genus *Bradyrhizobium*.

Strains 1.29Lᵀ, 1.27L and 5.13L exhibited traits associated with plant growth promotion and bioremediation potential, particularly under metal-contaminated conditions (Table S3). All three strains tolerated elevated concentrations of heavy metals, including lead (100–1,000 µg ml^−1^), zinc (100–1,000 µg ml^−1^), copper (100–300 µg ml^−1^) and cadmium (10–30 µg ml^−1^), suggesting the involvement of metal sequestration, bioaccumulation and/or efflux mechanisms [54]. The strains produced IAA, which contributes to root development and enhanced nutrient uptake, particularly under abiotic stress conditions [55]. Phosphate solubilization activity was also detected, indicating a capacity to increase phosphorus availability in nutrient-poor soils. In addition, siderophore production was observed, facilitating iron acquisition and potentially contributing to plant health and pathogen suppression [56]. In contrast, no ACC deaminase activity was detected, indicating a limited ability to alleviate ethylene-mediated stress responses in plants [57].

### Genome mining and functional analysis

Genome mining of strain 1.29Lᵀ revealed the presence of multiple genes involved in IAA biosynthesis, primarily associated with the indole-3-pyruvate (*iorAB*), indole-3-acetonitrile (*nhA*) and tryptamine (*moaA*) pathways. In addition, the *trpABCF* gene cluster, involved in tryptophan biosynthesis, the principal precursor of IAA in bacteria, was identified [58] (Table S4). The presence of these genes is consistent with the IAA production observed *in vitro* for strain 1.29Lᵀ.

Strain 1.29Lᵀ also demonstrated siderophore production *in vitro*, which was supported by genomic predictions of three biosynthetic gene clusters encoding the siderophores potashchelin A–D, azotobactin D and xenotetrapeptide (Fig. S4). In addition, several genes encoding siderophore and ferric iron transport systems were identified, including *feoAC*, *fhuAC* and *exbB*, which are essential for iron acquisition and bacterial fitness under iron-limited conditions [59].

Genes involved in phosphate solubilization were also detected in the genome of strain 1.29Lᵀ. These included *gcd* and the *pqqBDE* gene cluster, which mediate pyrroloquinoline quinone-dependent gluconic acid production, a major mechanism of inorganic phosphate solubilization by soil bacteria [60]. Additional genetic determinants associated with phosphate metabolism were identified, including the *phnABFGHIJKLNOP* and *phoABHQRU* operons, the high-affinity phosphate transport system *pstABCS* and the genes *ppa*, *ppk* and *ppx*, which are involved in phosphate mobilization, transport and intracellular storage (Table S4).

Consistent with the observed phenotypic tolerance to heavy metals, the genome of strain 1.29Lᵀ contained numerous genes associated with metal resistance, transport and homeostasis. These included genes involved in zinc uptake and regulation (*znuABD* and *zur*), copper resistance and trafficking (*cutE*, *copD*, *cueO*, *sco1* and *cox17*), arsenic resistance (*arsBC*), magnesium and cobalt transport (*corAC*) and resistance to multiple metals, including lead, cobalt, cadmium, nickel and mercury (*czcAD* and *zntA*) (Table S4). Collectively, these genomic features corroborate the strong metal tolerance and plant growth-promoting traits observed for strain 1.29Lᵀ.

## Description of *Bradyrhizobium cedriense* sp. nov.

**Etymology:** *Bradyrhizobium cedriense* sp. nov. (ce.dri.en’se. N.L. neut. adj. *cedriense*, referring to Borj-Cedria, Tunisia, the site of isolation of the type strain).

Cells are Gram-negative, aerobic, motile, non-spore-forming rods. Colonies on YMA are circular, whitish and less than 1 mm in diameter after 7 days of incubation at 28 °C. Optimal growth occurs at 28 °C, with growth observed at pH 6.0–9.0. Strains are salt-sensitive, with no growth above 0.8% (w/v) NaCl.

The type strain, 1.29Lᵀ, utilizes *α*-d-glucose, d-mannose, d-fructose, d-galactose, d-fucose, d-sorbitol, d-mannitol, glycerol, l-aspartic acid, l-pyroglutamic acid, d-galacturonic acid, l-galactonic acid lactone, d-gluconic acid, d-glucuronic acid, glucuronamide, mucic acid, d-saccharic acid, methyl pyruvate, *α*-ketoglutaric acid, Tween 40, *γ*-amino-butyric acid and *β*-hydroxy-d,l-butyric acid. The type strain grows in the presence of 1,000 µg ml⁻¹ Pb and Zn and 200 µg ml⁻¹ Cu. It is capable of solubilizing phosphate, producing siderophores and IAA, but does not exhibit ACC deaminase activity.

The type strain forms effective nodules on *A. saligna*. The major fatty acid is summed feature 8 (C_18 : 1_ ω7c), with notable amounts of C_18 : 2_ ω6 and C_16 : 0_.

The genome of the type strain 1.29Lᵀ is ~7,878,461 bp, with a G+C content of 63.8 mol%. Whole-genome sequences are available under NCBI GenBank accession numbers JBTSWB000000000 (1.29Lᵀ), JBTSWA000000000 (1.27L) and JBTSVZ000000000 (5.13L). The GenBank accession numbers for the 16S rRNA, *atp*D, *rec*A, *gln*II, *gyr*B and *nod*C genes of the type strain are OR091471, PX517230, PX517221, PX517224, PX517227 and PX557011, respectively.

The type strain 1.29Lᵀ (=LMG 33169ᵀ=DSM 116455ᵀ) was isolated from an effective nodule of *A. saligna* growing in soil from Borj Cedria, northeastern Tunisia.

## Supplementary material

10.1099/ijsem.0.007127 Fig. S1.

## Abbreviations

ACC: 1-aminocyclopropane-1-carboxylate
ANI: average nucleotide identity
dDDH: digital DNA–DNA hybridization
GTDB: Genome Taxonomy Database
IAA: indole-3-acetic acid
MAGs: metagenome-assembled genomes
ML: maximum-likelihood
MLSA: multilocus sequence analysis
TY: tryptone–yeast extract
TYGS: Type Strain Genome Server
YEM: yeast extract mannitol
YMA: yeast mannitol agar
