# Supplementary material for: Bradyrhizobium cedriense sp. nov., a novel rhizobial species isolated from Acacia saligna nodules grown in polluted soils in Tunisia
Source: Int J Syst Evol Microbiol. 2026 Apr 10;76(4):007127. doi: 10.1099/ijsem.0.007127 (PMC13068376; doi:10.1099/ijsem.0.007127)

Supplementary tables

Table S1. List of the 116 conserved single-copy marker genes used for GTDB-based phylogenomic analysis (Excel file attached).

Table S2. FastANI values between strain 1.29L^{T} and 1,412 reference genomes (A) and 329 metagenome-assembled genomes (MAGs) (B) of the genus *Bradyrhizobium* (Excel file attached).

Table S3. Heavy metal resistance and plant growth-promoting (PGP) traits of *Bradyrhizobium cedriense* sp. nov. strains 1.29L^{T}, 1.27L, and 5.13L isolated from *Acacia saligna* root nodules.

			1.29L^{T}	1.27L	5.13L
Heavy metals (in µg ml^{-1})	$PbCl_2$	100	+	+	+
		300	+	+	+
		500	+	+	+
		800	+	+	-
		1000	+	+	-
	$CdCl_2$	10	+	+	+
		20	+	+	+
		30	-	-	-
	$ZnCl_2$	100	+	+	+
		300	+	+	+
		500	+	+	+
		800	+	+	+
		1000	+	+	-
	$CuSO_4$	100	+	+	+
		200	+	+	+
		300	-	-	-
PGPR Activities	IAA production (µg/ml)		35.3^{a}	31.5^{b}	32.3^{b}
	Siderophore production (mm)		16.2^{a}	18.1^{a}	16.5^{a}
	ACC desaminase activity		nd	nd	nd
	Phosphate solubilization (mm)		13.1^{a}	15.5^{a}	14.2^{a}

Means (n = 5) with different lowercase letters in the same column are significantly different ($p \leq 0.05$, Tukey's HSD test); nd; not detected

Table S4. *In silico* prediction of genes involved in indole-3-acetic acid (IAA) and siderophore biosynthesis, phosphate solubilization, and heavy metal resistance in strain 1.29L^{T} (Excel file attached).

Supplementary figures

Figure S1. Maximum-likelihood phylogenetic analysis based on *rrs* gene (16S rRNA) sequences (1,215 nucleotides). The three novel strains are highlighted in bold. Bootstrap values ≥50% are shown at the corresponding nodes (based on 1,000 replicates). Each species name is followed by the strain designation and the NCBI accession number of the sequence used. The scale bar represents the number of substitutions per site.

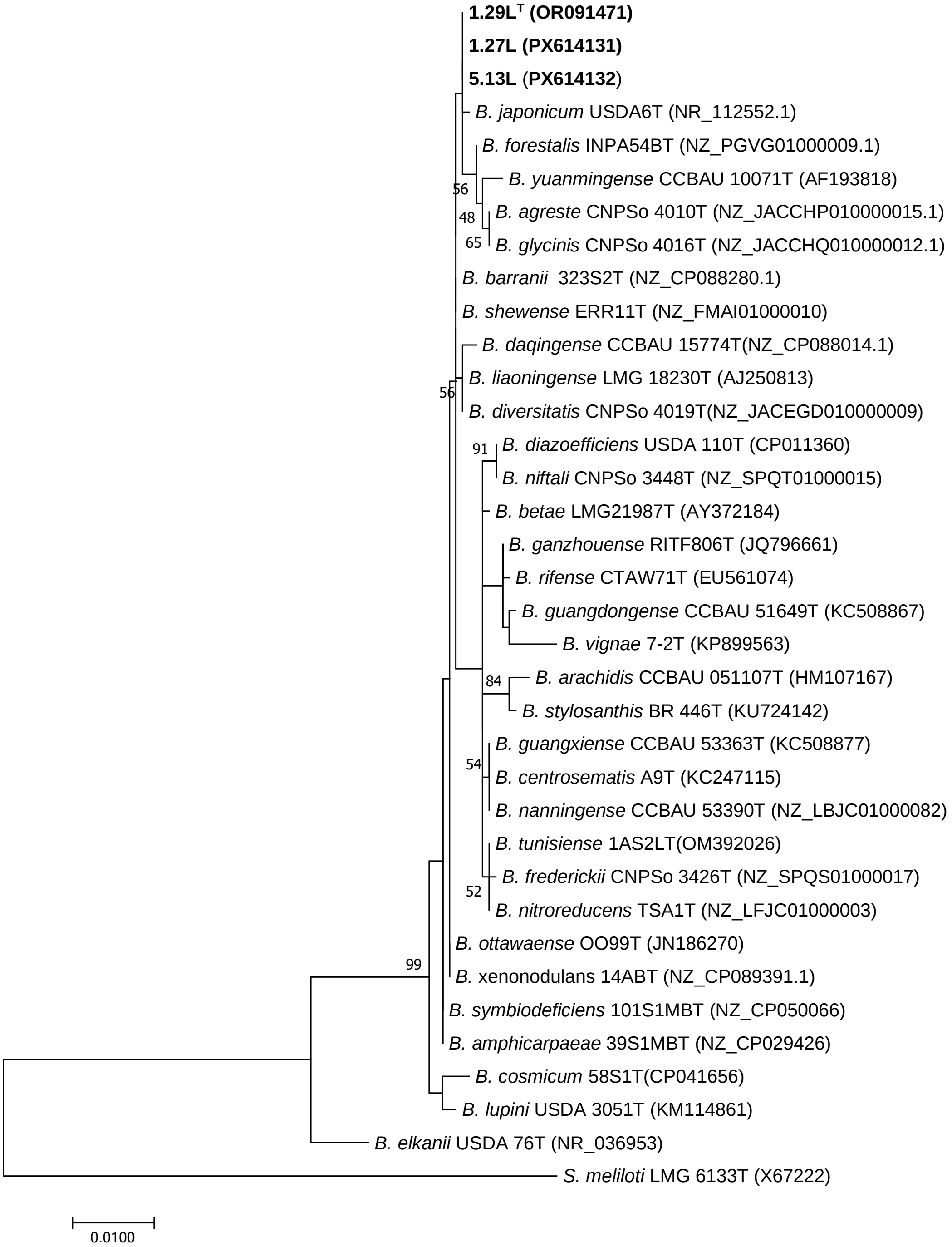

Figure S2. Maximum-likelihood phylogenetic analysis based on concatenated *atpD*, *recA*, *glnII*, and *gyrB* gene sequences (1,734 nucleotides). The three novel strains are indicated in bold. Bootstrap values ≥50% are shown at the corresponding nodes (based on 1,000 replicates). For each species, the strain designation and NCBI accession number of the sequences used are provided. The scale bar represents the number of substitutions per site.

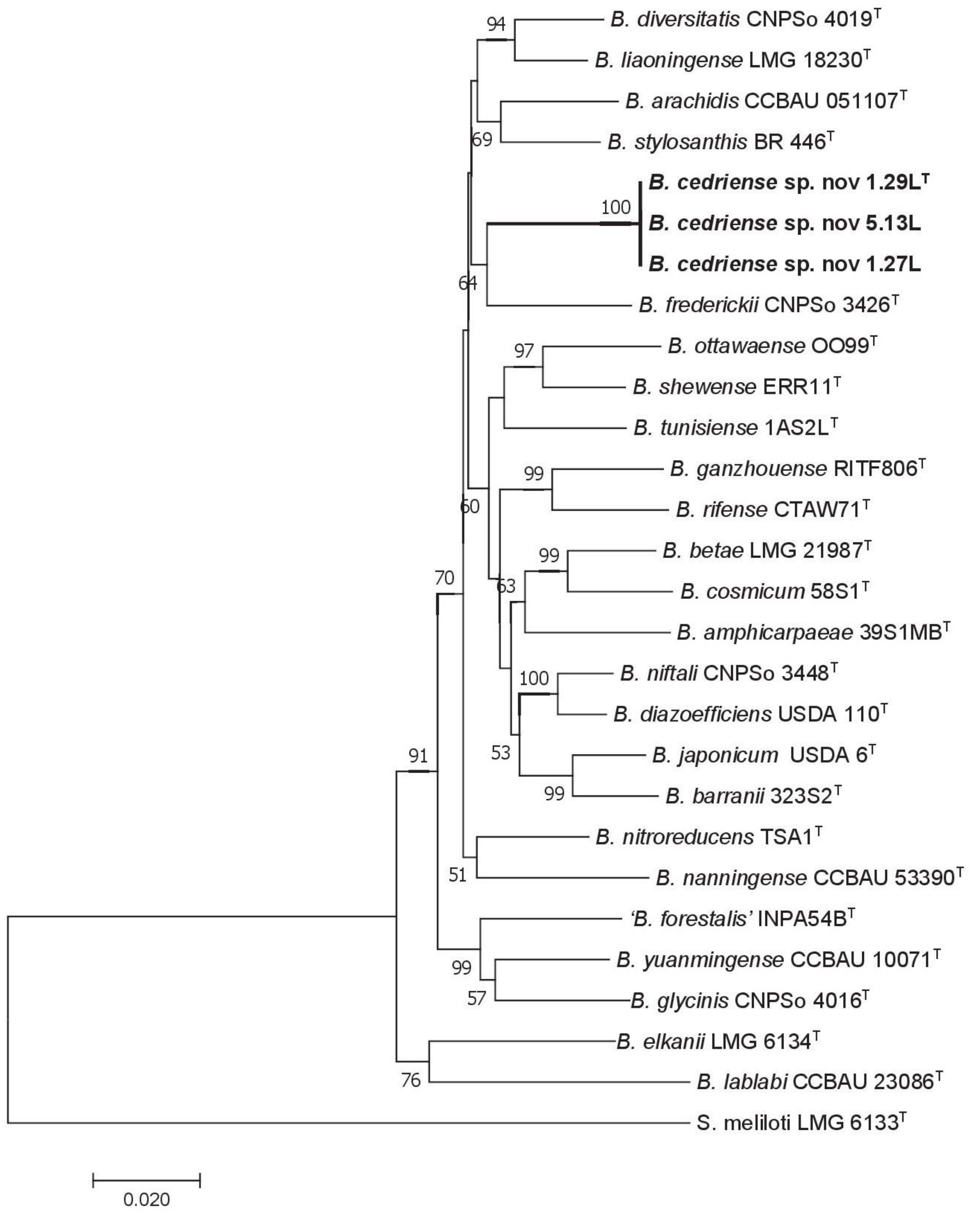

Figure S3. Transmission electron microscopy (TEM) image of the type strain 1.29L^T of *Bradyrhizobium cedriense* sp. nov.

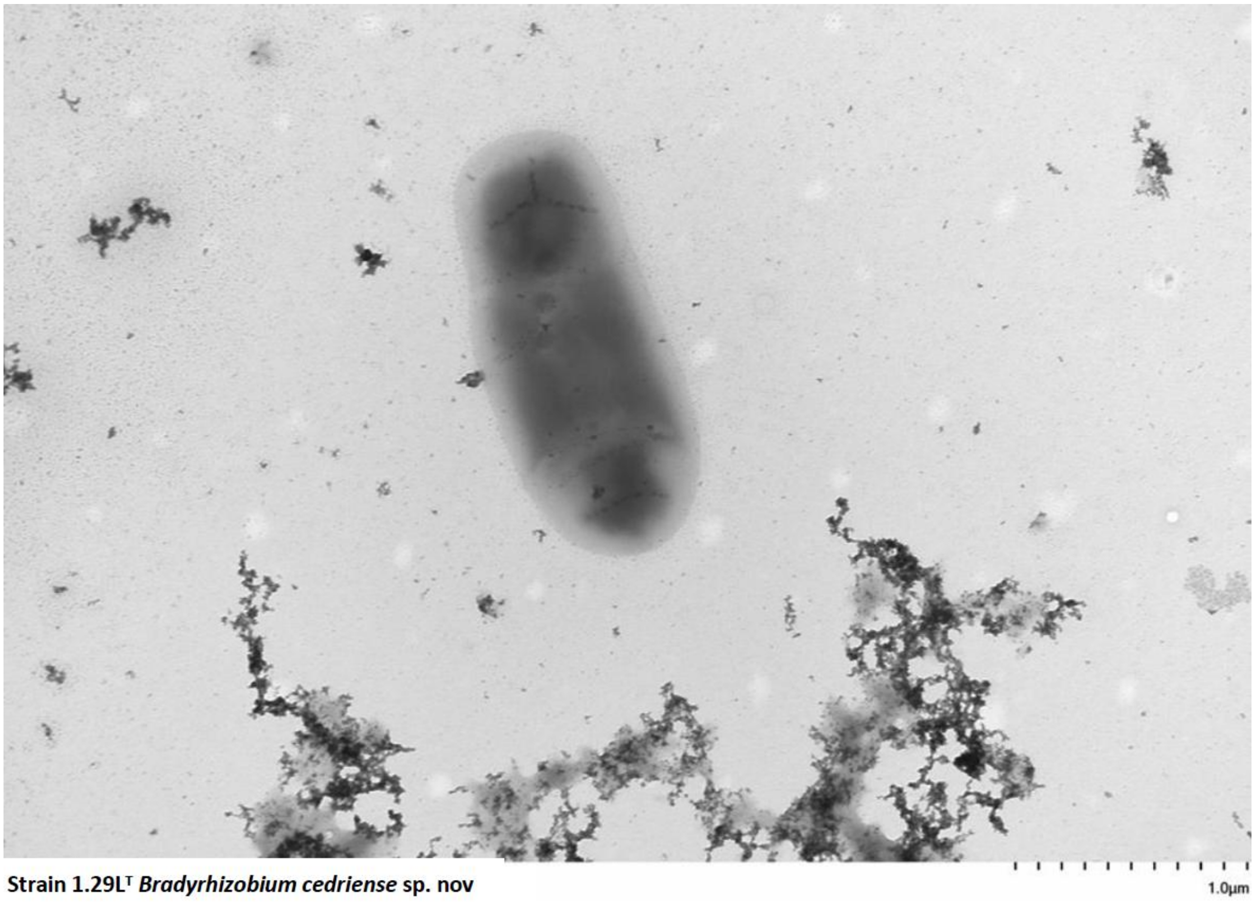

Figure S4. Genomic organization of biosynthetic gene clusters involved in the production of the siderophores potashchelin A-D, azotobactin D, and xenotetrapeptide in strain 1.29L^{T}.

Potashchelin A/potashchelin B/potashchelin C/potashchelin D

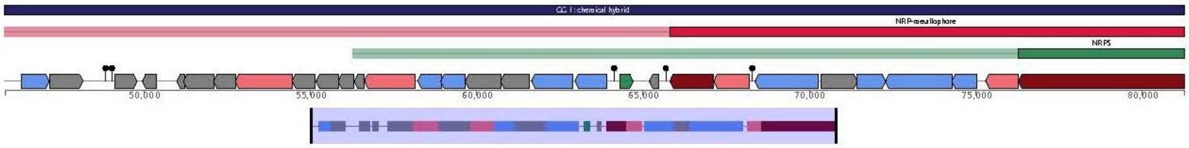

Azotobactin D

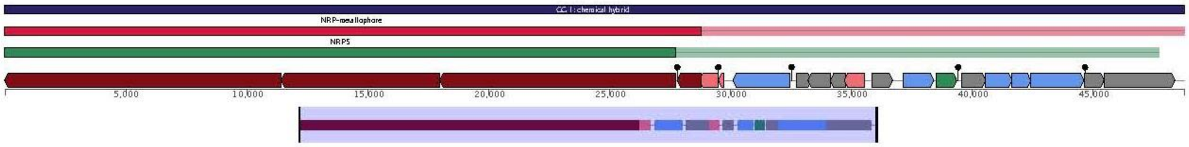

Xenotetrapeptide

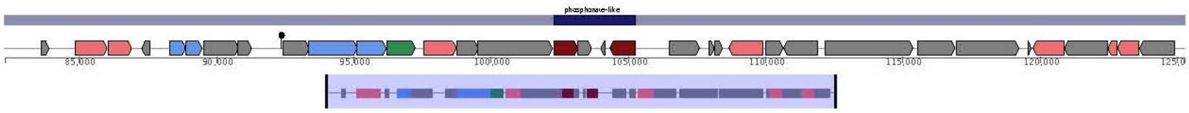

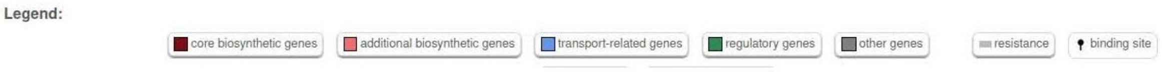

Supplement: Fig. S1. [file ijsem-76-07127-s001.pdf]
